# Challenges and Changes of Pharmacy Practice During the COVID-19 Crisis in Malaysia: Instability as an Opportunity

**DOI:** 10.21315/mjms2021.28.2.16

**Published:** 2021-04-21

**Authors:** Manvikram Singh Gill

**Affiliations:** Department of Pharmacy, Tuanku Mizan Armed Forces Hospital, Ministry of Defense, Wangsa Maju, Kuala Lumpur, Malaysia

**Keywords:** pharmacy practice, COVID-19, military hospital, Malaysia

## Abstract

Pharmacy practice is the discipline concerning the roles of pharmacists in the healthcare setting. Healthcare in Malaysia encompasses numerous sectors, such as academics, community, enforcement, hospital, industrial, jurisprudence, military, regulatory and research and development. In addressing the crisis brought on by the coronavirus disease 2019 (COVID-19) pandemic, pharmacists and technicians have been highly involved in the delivery of healthcare services. Malaysia has a distinct two-tier healthcare system and within this context, it is essential to integrate services during a crisis to maximise the available but limited medical resources. Often, the focus is on realistic and logical public–private partnerships. However, integrating different ministries offering healthcare facilities is also important and inter-agency pharmacy practice coordination must be optimised. The Malaysian Armed Forces Health Services can be primed to enhance the nation’s healthcare capacity and capability. As the COVID-19 pandemic continues to grip the nation and cause an unprecedented number of Malaysians to become very ill, pharmacists must be resilient in leading, adapting and integrating well-rounded strategies in their respective fields to ensure good pharmacy practice.

## Public Health Pharmacy Practice in Malaysia

Public health is the science of protecting and improving the health of the people and their communities (1). This is achieved by researching, detecting, preventing and responding to diseases (2). Public health is part of the people’s welfare. The barriers to a good public health framework include poor access to healthcare services, which may further be categorised systematically into man, machine, material, method and money. The dominant aspect of healthcare service is the provision of efficient and effective pharmaceutical support in which the pharmacy team plays a pivotal role. Pharmacy practice is the discipline concerning the roles of pharmacists in the healthcare setting. Meanwhile, the healthcare system in Malaysia encompasses numerous sectors, such as academics, community, enforcement, hospital, industrial, jurisprudence, military, regulatory and research and development (R&D). Pharmacists have an active role to play in all these sectors.

## Hospital Pharmacy Practice During Usual Times in Malaysia

Malaysia’s existing healthcare system consists of a two-tier public–private system. The private system mainly caters to the urban population and those who have the means to pay for their healthcare services. The public system provides access to everyone with token payments being imposed (3). Government-managed healthcare facilities are under the supervision of the Ministry of Health (MOH), the Ministry of Higher Education (MOHE) or the Ministry of Defence (MOD). Regardless of whether a system is public or private, pharmacists and pharmacy technicians are vital in providing patient-centred care in hospital complexes. Generally, in a hospital, the pharmacy department may be further divided into three major divisions: the inpatient pharmacy, the out-patient pharmacy and the medical store. The services provided by each division vary.

The principal role of the in-patient pharmacy is to supply medications to warded patients (also known as drug distribution activities) via multiple mechanisms, such as the unit dose system (UDS) and ward floor stock. Other specific in-patient care services provided are clinical pharmaceutical care, pharmacokinetics (also known as therapeutic drug monitoring or TDM), parenteral nutrition (PN), cytotoxic drug reconstitution (CDR), drug information services (DIS), extemporaneous preparations and galenical preparations (4).

The principal role of the out-patient pharmacy is to dispense medications to discharged in-patients or to out-patients who consult a doctor at the hospital clinics. This objective may be achieved by multiple mechanisms. The main mechanism involves requiring the patients to physically come and collect their medications monthly. The pharmacy appointment duration may differ according to the specific hospital, as this depends on the stock of the medications provided to the patients during each visit. There are also multiple value-added service (VAS) mechanisms offered to refill prescription medications. Various forms of VAS, such as medications postal/courier services, automated dispensing units and drive-through pharmacies, are offered to minimise the number of people going to pharmacies, thereby reducing patients’ waiting time at such places. The VAS alternatives offered by a hospital to its patients may differ according to the facility’s suitability and the patients’ medications. For instance, not all medications can be delivered by courier services, especially cold-chain and liquid dosage medications. Another example of a specific patient care service provided in this setting is the medication therapy adherence clinic (MTAC). Furthermore, both in-patient and out-patient pharmacies provide medication counselling. Medication counselling is a huge aspect of pharmacotherapy and encompasses all aspects of a medication review or reconciliation session.

Meanwhile, the main role of the DIS is to support clinical pharmacy services by responding to queries from healthcare providers and the public, developing guidelines, conducting pharmacy and therapeutic committee activities, involvement in the coordination of audits, educational activities, supporting publications, performing studies and reporting adverse drug reactions (ADR) or medication errors (ME), and performing drug use evaluations (DUEs) and drug use reviews (DURs) of medications in the hospital formulary or medication samples (i.e. investigational drug control).

The principal role of the medical store is to oversee the supply chain management (SCM) of the hospital’s pharmaceutical and medical logistics (PharmaMedLog). This encompasses the planning and management of all activities involved in sourcing and procurement, conversion and all healthcare-related logistics management activities. Importantly, it also includes coordination and collaboration with channel partners, which can include suppliers, intermediaries, third party service providers and customers (5). In essence, SCM integrates supply and demand management within and across hospital systems.

## Hospital Pharmacy Practice During a Crisis in Malaysia

In December 2019, a novel coronavirus (severe acute respiratory syndrome coronavirus-2 or SARS-CoV-2) has caused an outbreak of the novel coronavirus disease 2019 (COVID-19), starting in Wuhan, China and spreading quickly to the rest of the Mainland and to over 190 countries worldwide (6). As of March 2020, more than 700,000 confirmed cases of COVID-19 and 30,000 related deaths had been reported worldwide. Europe and the USA have since replaced China as the epicentre of the disease, with several countries (including Italy, Spain and Germany) reporting rapidly growing cases.

During the COVID-19 pandemic, the war analogy of ‘frontliners’ is being used to represent assistant medical officers, doctors (regardless of whether they function as housemen, medical officers, specialists, registrars or consultants), lab technicians and nurses. There are some instances wherein the commitment of the pharmacists and pharmacy technicians is overlooked. However, it must be emphasised that pharmacists and pharmacy technicians are very much involved from the beginning to end in the delivery of healthcare services during the COVID-19 crisis. In particular, they function behind the scenes from the community to the hospital levels.

## Customising Healthcare Services According to the Crisis Whilst Maximising the Impact of Available Resources

Healthcare services for the management of COVID-19 begin at the medical store. Here, the PharmaMedLog pharmacist on duty ensures the procurement of protective gears as a defence mechanism to safeguard healthcare workers involved directly and indirectly in managing the crisis. The items may include assets, such as non-contact infrared (IR) forehead thermometer, battery powered air-purifying respirators, medical disposables, hand sanitisers and personal protective equipment (PPE). The forms of PPE include Tyvek suits, 3-ply surgical or medical masks (ear loop or tie on), respirator masks (medical or particulate), plastic aprons, surgical/isolation gowns, disposable gloves, leg covers, shoe covers, waterproof boots, face shield, goggles or eye-visors, head covers and medical cap. All of these are fast-moving items in the current scenario. Apart from the supply of these items, the PharmaMedLog pharmacist has to ensure that medications are available to treat positive COVID-19 cases and patients under investigation. As a foresight, it is essential to stock up on COVID-19 treatment drugs, including hydroxychloroquine (Plaquenil), azithromycin (Zithromax), oseltamivir (Tamiflu), lopinavir/ritonavir (Kaletra), favipiravir (Avigan), remdesivir, tocilizumab (Actemra) and interferon beta-1b (Betaferon). Meanwhile, the PharmaMedLog procurement for non-COVID-19 cases continues routinely. The pharmacist managing the galenical services may also stock up on alcohol-based hand sanitisers. Mass self-production is done to address shortages, cater to increased demands and reduce costs.

The Malaysian government announced a Movement Control Order (MCO) for two weeks to be executed initially from 18 March 2020 to 31 March 2020. By May 2020, the MCO was extended until 6 June 2020 (7). Enhanced MCO was implemented in identified COVID-19 hotspots. Since this announcement, non-essential clinic appointments have been re-scheduled. This step guarantees minimal unnecessary patient traffic at hospitals to ensure that social distancing is being applied and healthcare human resources can be directed towards the management of COVID-19 cases. However, such a move has affected out-patients who are supposed to see their doctors or refill medications from the pharmacy during this period. In this case, good pharmacy practice has to be observed, as medication compliance is of utmost importance even during crisis to minimise the risk of patient hospitalisation. There should be no instance wherein patients must risk themselves to COVID-19 exposure just to get their medications; moreover, patients should not skip their medications doses due to insufficient supply. Recognising the risks, most out-patient pharmacy teams throughout the country have taken strategic measures to make it easier for people to get their medications even during the pandemic.

The prescriptions received by out-patient pharmacy teams can be categorised into two: medication filling of a new prescription or medication refilling of an existing prescription. For essential cases, the doctor may still see the patient at a clinic and issue a new prescription. The outpatient-pharmacy team will fill this prescription according to the normal pathway wherein the patient is present physically. However, instead of the usual 1- or 2-month supply of medications, the patients are supplied with 3 months’ worth of medications, thus limiting the number of trips that must be made. For non-essential cases with re-scheduled appointments, the doctor will do a tele-medicine session with the patient, write a prescription online and the clinic staff will send it to the pharmacy. The out-patient pharmacy team will then analyse the case during the screening of prescription. There will be two scenarios. If there are no changes in the patient’s pharmacotherapy, then the medications will be supplied via VAS and the patient will no longer be required to come to the pharmacy. If there are changes in the pharmacotherapy, then the prescription will be filled in advance and the patient will be advised as to when he/she should visit the pharmacy for a scheduled medication counselling and collection session. This depends on a case-by-case basis rationalisation by the pharmacy team.

All patients who request for the medication refilling of an existing prescription are advised to use VAS, although they may still physically come to the pharmacy to collect their medications. The VAS is promoted through various channels, especially via social applications like WhatsApp and Facebook. Patients need not be present physically to request for these services. Instead, they may call the pharmacy and arrange for such services. However, in cases wherein patients must be physically present in the pharmacy, it is vital to ensure social distancing in the waiting area of the out-patient pharmacy in order to limit their potential exposure to other patients and even the staff. The seats and pharmacy floors are properly labelled to enforce social distancing. Patients are advised to avoid sitting and standing close to one another whilst maintaining a distance of at least 1 metre apart. They are also advised to maintain distance with the pharmacy team when handing in their prescription at the prescription screening counter and use the hand sanitisers provided at the prescription screening counters. Information on COVID-19 and notices on the importance of hand hygiene are prominently displayed throughout the area. Safely refilling prescription medications during the COVID-19 pandemic takes some planning. All these strategies help to protect vulnerable patients from unnecessary risks whilst ensuring that they get the medications they need to stay healthy and out of the hospital.

Meanwhile, the in-patient pharmacy team in a non-COVID-19 treatment facility may face different challenges compared to the in-patient pharmacy team in a COVID-19 treatment facility. Although a hospital may not be treating COVID-19 cases, it should still have wards for quarantine cases. The in-patient pharmacy team has to ensure that these additional wards are adequately supplied with the required medications via available mechanisms. It is quite challenging to perform the normal functioning of all ward drug distribution mechanisms whilst practicing greater caution to ensure minimal exposure between the ward team and pharmacy team. Normal work culture has to be practiced cautiously. Contact caution is also advised for all pharmacists and technicians managing other services for in-patients. Moreover, the pharmacy team must take the necessary precautions for themselves. This includes disinfecting their working areas daily, using proper PPEs and taking hazard leaves on rotational basis to minimise contact. During this pandemic, the primary task of the DIS is to perform COVID-19 information dissemination to healthcare providers and patients. Sharing relevant and accurate information from a pharmacist’s point of view is vital for the management of COVID-19.

## Integration of Pharmacy Practices Between the MOH and the MOD

Malaysia has a distinct healthcare system and integrating a wide range of related services during a crisis is essential in maximising available but limited medical resources (8). Often, the focus is on having a realistic and logical public–private partnership. However, integrating different ministries offering healthcare facilities (i.e. MOH, MOHE and MOD) is vital and inter-agency pharmacy practice coordination must be optimised.

The Malaysian Armed Forces Health Services (MAFHS) can be primed to enhance the nation’s healthcare capacity and capability. The MAFHS under the MOD are offered by the Royal Medical and Dental Corps (RMDC). The RDMC is categorised as a service support corps and serves in three branches: army, air force and navy. The RMDC has major and minor medical units. The major medical units are under the command of the MAF Head Quarters (MAFHQ) and the minor medical units are commanded by each service, respectively. The major medical units consist of the following: i) 91 Institute of Aviation Medicine; ii) 92 Institute of Underwater and Hyperbaric Medicine; iii) 93 AF Medical and Dental Depot; iv) 94 Terendak AF Hospital; v) 95 Tuanku Mizan AF Hospital (TMAFH); vi) 96 Lumut AF Hospital; vii) 97 Gemas Army Hospital and viii) 98 Wilayah Kota Kinabalu AF Hospital. The minor medical units consist of the: i) AF medical battalions; ii) AF sick quarters; iii) AF medical centres and iv) AF dental centres. The TMAFH is located in Wangsa Maju, Kuala Lumpur. The TMAFH lab is designated as a COVID-19 detection and validation facility. Hence, the TMAFH assists the MOH by running samples from all Titiwangsa District healthcare facilities and occasionally from the National Public Health Laboratory, Sungai Buloh.

As of April 2020, the designated COVID-19 treatment facilities in Kuala Lumpur include the following: i) Hospital Kuala Lumpur (HKL); ii) Hospital Sungai Buloh (HSB) and iii) University Malaya Medical Centre (UMMC). Both HKL and HSB are under the jurisdiction of the MOH whilst the UMMC is under MOHE. The administrative teams of the HKL and TMAFH have come up with a consensus on integrating patient care. All patients who come to the TMAFH will have to undergo screening and testing via oropharyngeal/nasopharyngeal swabbing at its Emergency Department (ED). The stable and non-stable patients will be segregated and admitted to the respective quarantine wards pending the results. If a patient is positive, then he/she is transferred from the TMAFH to the HKL for treatment. If negative, the patient is treated in the TMAFH accordingly. Walk-in patients who arrive at the ED complaining of COVID-19 symptoms, such as fever, runny nose and cough, are also subjected to the screening procedure. Patients who show signs and symptoms of influenza-like illness undergo swabbing and are then quarantined. They will be diagnosed as having severe acute respiratory infection/pneumonia to rule out COVID-19. With targeted testing, there is no basis to swab patients who do not meet the diagnosis criteria. Instead, the patient will be referred to the fever clinic set up in the ED. The patient will be managed accordingly and discharged with proper medications. Most of the patients who fall under this category are diagnosed as having an upper respiratory tract illness. Instead of collecting their medications from the ED pharmacy dispensary, the medications will be dispensed by a pharmacy technician on site. Reciprocally, non-COVID-19 HKL patients who need urgent treatment and care will be referred to the TMAFH for management according to specialty. This will enable the HKL team to focus on combating COVID-19 without jeopardising other patients.

With the implementation of the MCO, pharmacy policies at the TMAFH are also loosened up. The out-patient pharmacy refills the prescription medications for follow-up patients who are under the MOH healthcare facilities but are unable to visit these clinics to refill their medications due to valid reasons and are facing depletion of stock. This policy was finalised upon receiving a few requests from walk-in patients from various places, such as Sintok, Kedah; Temerloh, Pahang; and even from Papar, Sabah; who have medical follow-up appointments. These patients were mostly visiting their families serving with the MOD or were in Kuala Lumpur for business purposes but were unable to return immediately. Their medications will be refilled if they can prove and re-confirm their prescriptions. If there are any queries or doubts about the patients’ medications, further reconfirmation will be carried out with the medical team at their respective healthcare facilities.

## Conclusion

### Strengthening Resilience of the Malaysian Healthcare System

Based on the Registration of Pharmacist Act 1951, there are currently 19,454 registered pharmacists in Malaysia (9), indicating a 1:1,645 ratio of pharmacist to Malaysian citizens. As the COVID-19 pandemic continues to grip the nation and cause an unprecedented number of Malaysians to become very ill, pharmacists must be resilient in leading, adopting and integrating well-rounded strategies in their respective practices. If pharmacy teams are able to cope with the dynamic changes during a crisis to ensure good pharmacy practice, then they are able to win the hearts and minds of other healthcare providers and the general public. The current healthcare instability crisis in Malaysia due to COVID-19 is an opportunity for establishing the omnipresence of the pharmacy profession.
